# Mitochondrial Dysfunction in Autism and Attention-Deficit/Hyperactivity Disorder: Evidence from Genetic, Biochemical, and Neuroimaging Approaches

**DOI:** 10.3390/antiox15060764

**Published:** 2026-06-18

**Authors:** Tina R. Ram, Chunlong Mu, Sarah J. MacEachern, Jane Shearer

**Affiliations:** 1 Department of Biochemistry and Molecular Biology, Cumming School of Medicine, Faculty of Kinesiology, University of Calgary, Calgary, AB T2N 4N1, Canada; 2 Alberta Children’s Hospital Research Institute, University of Calgary, Calgary, AB T2N 4N1, Canada; 3 Hotchkiss Brain Institute, University of Calgary, Calgary, AB T2N 4N1, Canada; 4 Department of Microbiology, Immunology, and Infectious Diseases, Cumming School of Medicine, University of Calgary, Calgary, AB T2N 4N1, Canada; 5 Department of Pediatrics, Cumming School of Medicine, University of Calgary, Calgary, AB T2N 4N1, Canada

**Keywords:** mitochondria, oxidative stress, neurodevelopment, energy, phenotype, electron transport chain, neuroinflammation, bioenergetics, metabolism

## Abstract

Mitochondrial dysfunction has been increasingly implicated in the pathobiology of neurodevelopmental conditions, particularly autism and attention-deficit/hyperactivity disorder (ADHD). Because the developing brain is critically dependent on sustained ATP production, impairments in oxidative phosphorylation, mitochondrial dynamics, and redox balance may disrupt neuronal maturation, synaptic development, and neural circuit refinement during sensitive developmental periods. This review examines evidence from postmortem neurochemistry, genomics, magnetic resonance spectroscopy, and biomarker research to characterize mitochondrial impairment across autism and ADHD. Studies in autism report an elevated burden of heteroplasmic mitochondrial DNA (mtDNA) variants, along with alterations in mtDNA copy number, respiratory chain capacity, fission–fusion dynamics, and antioxidant defenses. Postmortem data demonstrate reduced activity of electron transport chain Complexes I, III, and V in the frontal cortex, temporal lobe, and cerebellum. These bioenergetic abnormalities are accompanied by elevated oxidative stress markers alongside mitochondria-mediated immune activation. *In vivo* neuroimaging corroborates these findings through elevated cerebral lactate and reduced phosphocreatine-to-ATP ratios. Evidence in ADHD is limited, but similarly implicates mitochondrial dysfunction, consistent with the frequent co-occurrence of these conditions and their partially shared architecture. The available literature supports mitochondrial dysfunction as a transdiagnostic biological feature of neurodevelopmental conditions, with relevance to mechanistic biomarker identification and targeted therapeutic development.

## 1. Introduction

Mitochondria are critical organelles governing ATP production and redox homeostasis. These processes are essential for brain development, including synaptic formation, neurotransmission, and neuronal plasticity. Given the high energetic demands of the developing brain, even subtle impairments in mitochondrial function can disrupt neurodevelopmental trajectories. Increasing evidence implicates mitochondrial dysfunction as a contributing mechanism in neurodevelopmental conditions, particularly autism and ADHD. These conditions frequently co-occur and can share genetic, metabolic, and neurobiological characteristics, suggesting the presence of similar underlying mechanisms.

Research over the past three decades has identified alterations in metabolism, mtDNA, respiratory chain activity, and oxidative stress markers in individuals with autism. Similar, though more limited, findings are emerging in ADHD, including disruptions in ATP production and mitochondrial dynamics. In both conditions, mitochondrial dysfunction is closely linked to increased oxidative stress, redox imbalance, and neuroinflammatory pathways, highlighting a broader mitochondrial contribution to brain development and function. Despite this, the literature remains fragmented across disciplines, with findings often considered in isolation.

The aim of this narrative review is to synthesize current evidence on mitochondrial dysfunction across autism and ADHD, drawing from genetic studies, biochemical analyses, neuroimaging, and clinical observations. We identify areas of both convergence and divergence and frame mitochondrial dysfunction as a cross-cutting biological process rather than a feature confined to individual diagnostic categories. This perspective may inform the development of targeted therapeutics focused on bioenergetic and mitochondrial pathways. The review was prepared in accordance with the Scale for the Assessment of Narrative Review Articles (SANRA) guidelines [1]. A comprehensive literature search was conducted using PubMed and Google Scholar for articles published from 1985 to 2025. Historical articles on metabolic findings in autism and ADHD are included for context and to show the progression of the field. Details of the search strategy can be found in the Supplementary Information.

## 2. Mitochondrial Physiology

Mitochondria generate cellular energy through oxidative phosphorylation along the inner mitochondrial membrane, where the electron transport chain (ETC), a series of enzymatic complexes, transfers electrons and establishes a proton gradient to drive ATP synthesis. Mitochondria contain their own circular genome of approximately 16.6 kilobases, which encodes 13 proteins essential for oxidative phosphorylation, along with transfer RNAs and ribosomal RNAs needed for mitochondrial protein synthesis [2,3,4]. Most mitochondrial proteins are encoded in the nuclear genome and imported into the organelle. This system requires precise coordination between nuclear DNA (nDNA) and mtDNA, and disruptions in either genome can compromise respiratory chain function. mtDNA is particularly vulnerable to damage as it is located near the main sites of reactive oxygen species production and lacks protective histones. Furthermore, mtDNA has limited repair capacity compared with nDNA [5]. These features make mtDNA susceptible to mutations, deletions and altered copy number, all of which can affect mitochondrial performance, hence impacting neurodevelopmental trajectories.

**Figure 1:**
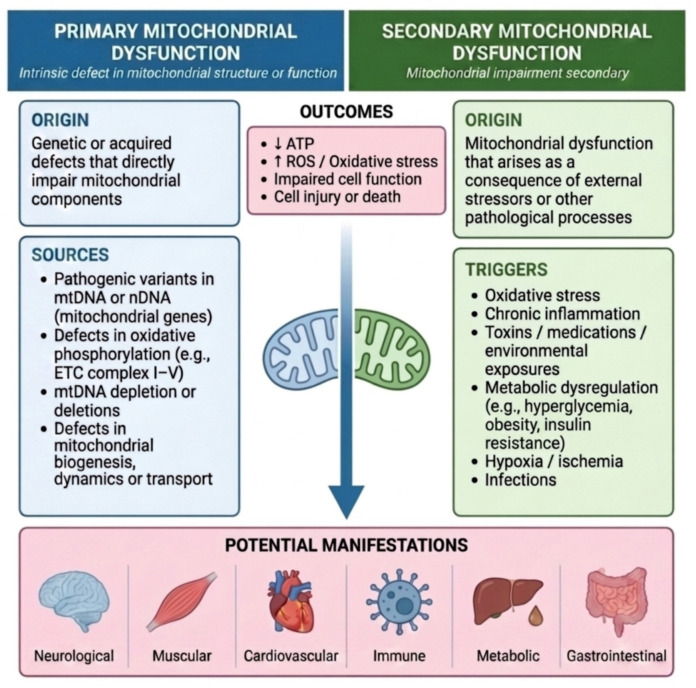
Primary versus secondary mitochondrial dysfunction. Primary mitochondrial dysfunction arises from intrinsic genetic or acquired defects directly impairing mitochondrial components, including pathogenic variants. Secondary mitochondrial dysfunction results from mitochondrial impairment consequent to external stressors, pathological processes or chronic conditions. Both primary and secondary dysfunction converge on common pathophysiological outcomes. These can manifest across multiple organ systems. Created in BioRender (2026). https://BioRender.com/2mqdrcn (accessed on 15 April 2026).

Mitochondrial dysfunction can be classified as either primary or secondary (Figure 1). Primary mitochondrial dysfunction arises from intrinsic defects in mitochondrial structure or function, typically due to pathogenic variants in genes encoded by mtDNA or nDNA that encode mitochondrial proteins, but also due to mtDNA depletion or deletions and impairments in mitochondrial biogenesis, dynamics, or transport [6]. In contrast, secondary mitochondrial dysfunction occurs as a consequence of chronic conditions, external stressors or pathological processes, including oxidative stress, chronic inflammation, toxin or medication exposure, metabolic dysregulation, hypoxia, and infection [7]. Secondary dysfunction has been documented in several neurodevelopmental conditions, including autism and ADHD [8,9], though whether this represents a cause or consequence of these disorders is unknown. While the origins differ, both primary and secondary mitochondrial dysfunction have common outcomes: decreased ATP production, increased reactive oxygen species generation, impaired cellular function, and potential cell injury or death [10]. The distinction between primary and secondary dysfunction has important implications for understanding disease mechanisms and developing targeted interventions.

A defining characteristic of mitochondrial genetics is heteroplasmy, which refers to the presence of both wild-type and mutant mtDNA within the same cell. The proportion of mutant molecules determines the degree of functional impairment [11,12,13]. Tissues with high energetic demands, such as the brain, are especially affected when the proportion of mutant mtDNA surpasses the threshold for dysfunction. This threshold can vary depending on the gene affected, the nature of the mutation and the metabolic requirements of the affected tissue.

**Figure 2:**
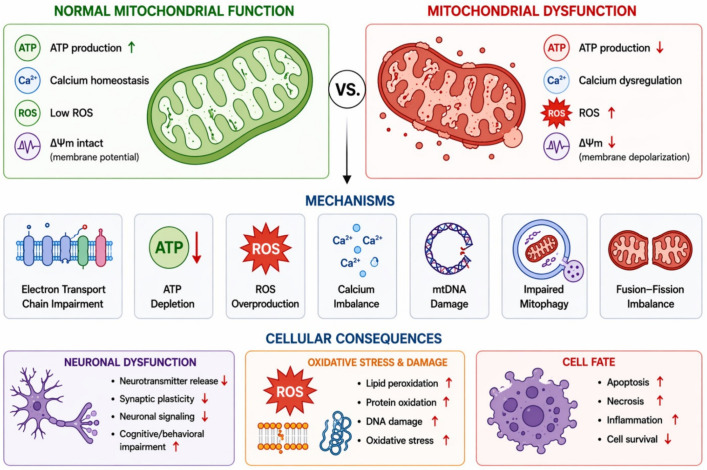
Comparison of normal and dysfunctional mitochondrial roles. A schematic comparing normal physiological mitochondrial function with mitochondrial dysfunction and associated cellular outcomes. Created in BioRender (2026). https://BioRender.com/0keg9y2 (accessed on 15 April 2026).

Beyond mitochondria’s hallmark role in the production of ATP, these organelles participate in several other cellular processes essential for neurodevelopment [14,15] (Figure 2). Mitochondria regulate intracellular calcium signaling and influence neurotransmitter release, neuronal excitability, and developmental patterning [16,17,18]. They also play a key role in apoptosis by releasing cytochrome c and other pro-apoptotic factors in response to cellular stress [19,20]. The maintenance of healthy mitochondrial populations is supported by dynamic processes such as fission, fusion, biogenesis, and mitophagy [21]. These mechanisms ensure the distribution of mitochondria during cell division, the mixing of mitochondrial contents to dilute damage, the removal of dysfunctional organelles, and the generation of new ones in response to metabolic demand. Dysfunction in these processes has been described in several neurodevelopmental disorders, including autism, and may contribute to altered neuronal connectivity and synaptic function [16,22,23,24,25].

## 3. Neurodevelopmental Conditions: Autism and ADHD

Autism is a neurodevelopmental condition characterized by differences in social communication, restricted or repetitive behaviors, and differences in sensory processing [26]. Although these features define the behavioral presentation and diagnostic criteria, autism is increasingly being understood as a biological condition influenced by both genetic and environmental factors [27,28,29,30]. The interplay of behavior and biology shapes a highly variable neurodevelopmental condition; heterogeneity is one of the defining features of autism today. Individuals vary widely in cognitive ability, language development, adaptive functioning, and co-occurring medical and psychiatric conditions [31,32,33]. This variability in presentation suggests that autism encompasses multiple biological subtypes, each shaped by a distinct makeup of genetic and physiological influences. Mitochondrial dysfunction has been hypothesized to be a subgroup within autism as many studies have reported a link between the biological features tied to mitochondrial dysfunction in autistic individuals [8]. the extent to which distinct mitochondrial profiles correspond to autism is unclear.

ADHD is a neurodevelopmental condition that is characterized by impairing patterns of inattention, hyperactivity, and/or impulsivity. It begins in childhood and influences daily functioning [26]. Similar to autism, studies have found that ADHD demonstrates high heritability, but environmental influences also play an important role [34,35,36,37]. Although research on mitochondrial function in ADHD is more limited compared with autism, several studies have begun to identify alterations in mitochondrial dynamics, ATP production, and redox balance [35,37,38].

**Figure 3:**
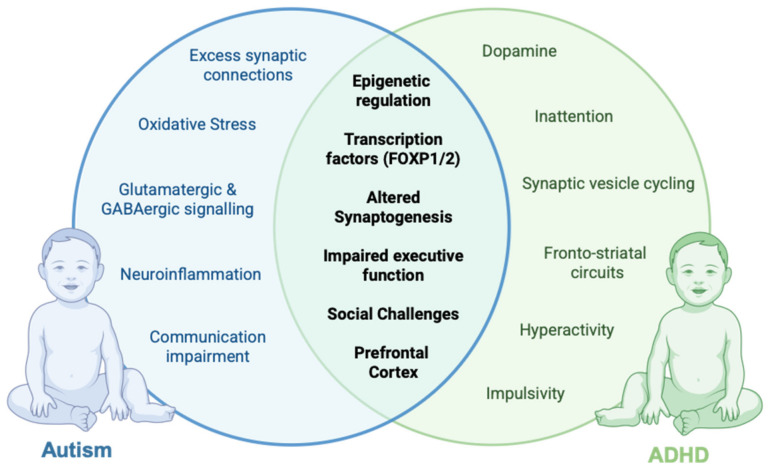
Overlap between autism and ADHD. This diagram illustrates the overlap and distinction between autism and ADHD in terms of genetic, neurobiological, and behavioral characteristics. The shared region highlights common features.Created in Biorender (2026). https://BioRender.com/ryals60 (accessed on 15 April 2026).

Autism and ADHD frequently co-occur [39]. Up to half of individuals with autism meet the criteria for ADHD, and many children with ADHD display traits associated with autism. Given the overlap between these conditions, there may be a possibility for shared biological mechanisms and underpinnings (Figure 3). Mitochondria present a reasonable candidate, as they regulate processes that influence attention, sensory processing, cognitive flexibility, sleep, and neuroimmune function [31,40]. This underscores the importance of studying mitochondrial function, not in relation to a single diagnosis but as a cross cutting biological dimension that may influence multiple neurodevelopmental outcomes, as reflected across pediatric cohorts reporting genomic, metabolic, and functional measures (Table 1).

## 4. Clinical and Intervention Challenges for Autism and ADHD

Understanding the biological factors associated with neurodevelopmental conditions is important for informing supportive strategies and guiding the development of targeted interventions aimed at relevant biological pathways. Autism presents considerable diagnostic and prognostic complexity due to substantial variation across cognitive, language, sensory, and behavioral domains. Current first-line supports for autistic individuals typically involve a multifaceted approach, including behavioral, speech, occupational, and play-based therapies, most commonly—and optimally—implemented during early childhood [14,37]. While no pharmacological agents have been developed to address the core features of autism, medications are frequently prescribed to manage co-occurring symptoms that may contribute to functional challenges, such as irritability, sleep disturbance, or attentional difficulties (e.g., aripiprazole, risperidone, melatonin, and stimulant medications) [87]. Early and individualized support strategies may contribute to improved functional outcomes and quality of life for autistic individuals.

ADHD presents with similar diagnostic and clinical complexity due to heterogeneity in symptom profiles, developmental trajectories, and co-occurring conditions [88]. At present, diagnosis relies on clinical assessment and structured informant reports, requiring the corroboration of impairing symptoms of inattention and/or hyperactivity–impulsivity across multiple settings [89]. First-line intervention approaches vary by age: behavioral and parent-training strategies are recommended as the primary intervention in young children, while pharmacotherapy is often recommended for school-aged children and older [89]. In North America, approximately 50–70% of children aged 3–17 years with a current ADHD diagnosis receive medication [90,91]. Pharmacotherapy includes stimulant medications such as methylphenidate and amphetamine formulations, as well as non-stimulant alternatives including atomoxetine, guanfacine, and clonidine, which are used when stimulants are not tolerated [89]. Despite the availability of effective pharmacological options, treatment response is variable, and multimodal approaches combining medication with behavioral and psychosocial interventions are generally recommended for individuals with moderate-to-severe impairment [92].

## 5. Genetic Evidence for Mitochondrial Involvement in Autism and ADHD

Advances in sequencing technologies have identified a growing number of mtDNA variants associated with autism, including heteroplasmic variants, copy number alterations, and changes in mitochondrial gene expression reported across pediatric neurodevelopmental cohorts (Table 1). Large cohort studies have reported that individuals with autism carry a higher burden of rare heteroplasmic mtDNA variants than typically developing controls [93,94]. Many of these variants affect protein coding regions, including genes responsible for components of the ETC [58,95]. Others occur in transfer RNA or ribosomal RNA genes, which can disrupt mitochondrial protein synthesis [93,94]. Although some variants reach high levels of heteroplasmy, even low-level heteroplasmic variants can impair respiratory chain activity, especially when combined with environmental stressors or nuclear genetic vulnerabilities [58].

Altered mtDNA copy numbers have also been observed in autism [4,96,97]. Some studies report an elevated copy number, which may represent a compensatory response to underlying mitochondrial dysfunction [98,99]. Others find a reduced copy number, suggesting impaired mitochondrial biogenesis or increased susceptibility to mtDNA damage [100]. Differences in study methodologies, tissue type, and age likely contribute to these discrepancies, but the overall pattern indicates that mtDNA regulation is disrupted in at least a subset of individuals with autism. The interplay between mitochondrial and nuclear genomes further complicates the genetic landscape. Many nuclear genes implicated in autism influence mitochondrial biology. These include genes that regulate respiratory chain assembly, mitochondrial transport, fission and fusion, antioxidant defenses, and apoptotic signaling [101]. Disruptions in nuclear genes can sensitize mitochondria to additional stressors or worsen the effects of mtDNA mutations [102]. Some studies provide evidence for synergistic interactions, where the combination of mtDNA variants and nuclear gene mutations produces more severe phenotypes than either alone [103,104]. This genetic interplay helps explain why individuals with similar mtDNA variants can exhibit different clinical presentations depending on their nuclear background.

While research on mitochondrial genetics in ADHD is limited, emerging studies suggest that similar processes may be involved. Some reports describe altered mtDNA copy number, an increased prevalence of mtDNA mutations, and changes in nuclear genes that regulate mitochondrial dynamics [105,106]. Although more work is required in this field, findings indicate that mitochondrial genetics may play a broader role across neurodevelopmental conditions.

## 6. Respiratory Chain Dysfunction in Autism and ADHD

Mitochondrial respiratory chain dysfunction refers to impairment in the electron transport chain that reduces the efficiency of oxidative phosphorylation, leading to altered cellular energy production and redox balance [7]. Mitochondrial respiratory chain dysfunction is reported in a considerable proportion of individuals with autism, ranging from 30 to 80%; this is considered one of the most replicated biological findings in this population [50]. The respiratory chain consists of enzymatic complexes that work in sequence to transfer electrons, pump protons across the mitochondrial inner membrane, and generate the electrochemical gradient needed for ATP synthesis [25,107]. Disruption in any of these complexes can diminish ATP production and increase the generation of reactive oxygen species (ROS), which places significant stress on metabolically active tissues [108,109]. Because the developing brain depends heavily on uninterrupted energy provision, impairments in the respiratory chain can alter neuronal maturation, weaken synaptic connectivity, and affect the refinement of neural circuits during early development [110,111,112].

Postmortem studies have provided some of the clearest evidence for respiratory chain impairment in autism. Investigations of the frontal cortex, temporal lobe, and cerebellum have consistently shown decreased activity of Complex I, which serves as the primary entry point for electrons into the respiratory chain [47,112,113]. Reductions in Complex III and Complex V activity have also been reported in some regions, suggesting that mitochondrial abnormalities may be both region-specific and developmentally sensitive [113,114,115]. Enzyme deficits appear most pronounced during childhood and early adolescence, suggesting that mitochondrial vulnerability may be especially important during periods of rapid synaptic development [43,47,115]. Several studies note a decline in the prominence of these abnormalities in adulthood, raising the possibility that some mitochondrial impairments are transient or that compensatory mechanisms emerge over time [116].

Peripheral tissues also reveal respiratory chain abnormalities in a substantial subset of individuals with autism. Buccal epithelial cell assays demonstrate that approximately forty percent of autistic children show reduced activity in one or more respiratory chain complexes [117]. These findings place mitochondrial dysfunction outside the brain, implicating a systemic rather than organ-specific process. The consistency of Complex I deficits across both central and peripheral tissues is particularly notable, as this complex is highly sensitive to genetic variation, oxidative stress, and inflammatory signals [43,101,113]. Lymphoblastoid studies provide further evidence by showing reduced Complex I activity and diminished pyruvate dehydrogenase function, which suggests broader disruption in oxidative metabolism [118]. An intriguing subset of individuals with autism displays a different pattern of mitochondrial function characterized by increased, rather than decreased, respiratory chain activity [101]. Taken together, these findings suggest that mitochondrial dysfunction in autism is heterogeneous and cannot be reduced to a single subtype of impairment.

Although fewer studies have examined respiratory chain activity in ADHD, emerging evidence suggests that similar bioenergetic alterations may be present. Some investigations report decreased ATP production, altered Complex I activity, and changes in mitochondrial coupling efficiency [9,119,120]. These abnormalities may contribute to the cognitive and behavioral features of ADHD, particularly deficits in sustained attention, variability in processing speed, and difficulties with executive functioning. Because the prefrontal cortex relies heavily on consistent energy for the regulation of attentional and inhibitory processes, even subtle impairments in mitochondrial function could influence ADHD symptoms [115,121,122].

## 7. Oxidative Stress and Redox Imbalance in Autism and ADHD

Elevated oxidative stress is a consistently reported biochemical marker in autism and has been closely linked to mitochondrial dysfunction. Oxidative stress arises when the production of ROS exceeds the capacity of cellular antioxidant systems to neutralize them [54,108,123]. Mitochondria are major sources of ROS, generated through electron leakage at Complexes I and III, and impaired respiratory chain function can magnify production. Accumulated reactive oxygen species damage lipids, proteins and nucleic acids, which can further impair mitochondrial function and disrupt cellular homeostasis [124,125]. Because the developing brain is particularly vulnerable to oxidative injury, even small increases in oxidative stress during critical periods can influence neurodevelopment [15].

Multiple studies have demonstrated elevated oxidative damage in the brains of autistic individuals. Postmortem analyses reveal increased levels of lipid hydroperoxides, which indicate peroxidation of neuronal membranes [126]. Elevated 3-nitrotyrosine and other markers of protein nitration suggest that reactive nitrogen species also contribute to tissue damage [127,128]. Oxidative damage to DNA is reflected by increased concentrations of 8-oxo-deoxyguanosine, which is a sensitive indicator of mitochondrial and nuclear DNA oxidation [129]. These abnormalities have been detected across several brain regions, including the temporal cortex and cerebellum, and appear to be particularly pronounced in younger individuals.

Peripheral evidence also aligns with these central findings. Several studies have reported decreased plasma levels of reduced glutathione, which is the most abundant intracellular antioxidant and serves as a key regulator of redox balance [52,53]. In addition, the ratio of reduced to oxidized glutathione is markedly lower in individuals with autism compared with typically developing controls, indicating a shift toward an oxidative state [53,130]. Enzymes involved in antioxidant defense, such as glutathione peroxidase and superoxide dismutase, are often reduced [50]. These systemic markers of oxidative stress suggest that redox imbalance is not confined to the central nervous system and likely reflects broader physiological vulnerability or stress.

Consistent with this, oxidative stress in ADHD has been linked to both neuroinflammation and mitochondrial dysfunction [74,86]. Reviews consistently report reduced antioxidant defenses and increased oxidative damage in ADHD [74,131]. A meta-analysis of 231 ADHD individuals and 207 controls found that ADHD individuals maintain normal antioxidant production but mount an insufficient response to oxidative challenge, resulting in net oxidative damage [77].

Of note, oxidative stress does not occur in isolation but interacts closely with mitochondrial dysfunction. Excessive reactive oxygen species damage mtDNA, respiratory chain proteins, and membrane lipids, which can reduce the efficiency of oxidative phosphorylation and further increase the production of reactive species. This creates a cycle in which mitochondrial dysfunction and oxidative stress reinforce one another. Because neurons depend on consistent and high-level ATP production, this cycle can have substantial consequences for neuronal development and function. Oxidative stress and mitochondrial impairment also influence synaptic plasticity, neurotransmitter regulation and calcium homeostasis, all of which are relevant to the behavioral features of autism and ADHD [108,132].

## 8. Neuroinflammation and Immune Mitochondrial Interactions

Evidence shows that neuroinflammation is a significant feature of autism and may also contribute to biological differences observed in ADHD. Neuroinflammation involves activation of microglia and astrocytes, which release cytokines and other mediators in response to injury, infection, or metabolic stress [133,134]. Although these processes support normal development, persistent activation can interfere with neuronal maturation, synaptic function, and network connectivity. Increasing data also indicate the existence of a close relationship between neuroinflammation and mitochondrial dysfunction, with each process contributing to ongoing cellular stress.

Postmortem studies in autistic individuals report widespread microglial activation in the cortex, cerebellum, and white matter [66,67]. Elevated interleukin and tumor necrosis factor signaling, along with altered microglial morphology, suggest chronic inflammatory activity across childhood and adulthood [135]. These patterns imply that neuroinflammation is a core characteristic of the autistic brain rather than a secondary response to environmental factors. Mitochondria play a central role in inflammatory signaling. During cellular stress, mitochondrial components such as mtDNA fragments and cardiolipin can enter the cytoplasm or extracellular space and activate innate immune receptors, including toll-like receptor 9 and the inflammasome [136]. This activation increases production of proinflammatory cytokines and heightens glial sensitivity, creating a reinforcing cycle of inflammation and mitochondrial injury. This interaction is particularly relevant in autism, where mitochondrial abnormalities frequently coincide with elevated inflammatory markers.

Neuroinflammation may also influence behavioral features. Microglia guide synaptic pruning, and prolonged activation can disrupt connectivity patterns commonly reported in autism [137]. Inflammatory activity also affects glutamate and gamma-aminobutyric acid systems, which regulate excitatory and inhibitory balance [67,137]. Altered signaling in these pathways has been linked to autism and ADHD and may contribute to sensory hypersensitivity, emotional dysregulation, and attentional variability [119,138].

For the most part, there is weak evidence for neuroinflammation in ADHD and most reports of elevated immune tone and dysregulation overlap with oxidative stress and mitochondrial dysfunction [74,86]. Evidence is largely derived from comorbidity studies showing above-chance, co-occurrence of ADHD with autoimmune and inflammatory disorders, reports of elevated serum cytokines, and polymorphisms in inflammation-related genes [85,86]. Findings are limited by small samples, biomarker heterogeneity, and a lack of tissue-level studies. At present, the neuroinflammation literature in ADHD is almost entirely derived from animal models [85]. There are no postmortem human brain studies characterizing glial cell density, morphology, or activation state in ADHD compared to controls. This constitutes a substantial evidence gap relative to autism, where both postmortem and *in vivo* imaging data on microglial activation exist.

## 9. Clinical Features Linked to Mitochondrial Dysfunction in Autism and ADHD

Clinical features associated with mitochondrial dysfunction in autism are diverse and often overlap with selected symptoms observed in primary mitochondrial disorders. Although most individuals with autism do not meet full diagnostic criteria for a classical mitochondrial disease, many display patterns of physiological impairment that resemble milder or more heterogeneous mitochondrial phenotypes [12,139]. These clinical features tend to cluster in systems with high energetic demands, particularly the brain, gastrointestinal tract, and muscular system [140]. The variability in presentation suggests that mitochondrial dysfunction contributes to a wide range of symptoms in autism and may underlie some of the heterogeneity that has historically complicated clinical assessment.

Fatigue and exercise intolerance are frequently reported in individuals with mitochondrial abnormalities. In autism, caregivers often describe lower endurance, reduced physical stamina, and increased susceptibility to physical or cognitive fatigue [141,142]. Although many studies rely on caregiver reports rather than direct physiological measurement, this consistent pattern mirrors the fatigue commonly seen in mitochondrial disorders. Musculoskeletal hypotonia is also common in autistic children and may reflect reduced ATP generation within muscle cells [143]. Low muscle tone can influence motor development, coordination, and posture, and it may contribute to the motor delays frequently observed in autism [144]. Some studies document elevated serum lactate and pyruvate levels in affected individuals, which are metabolic indicators of impaired oxidative phosphorylation [145,146]. Elevated lactate, particularly following mild exertion, supports the presence of underlying mitochondrial inefficiency [145].

Many of the clinical features seen in autistic children are also reported in children with ADHD. Children with ADHD often report chronic fatigue and reduced exercise tolerance [147,148]. Sleep–wake disturbances such as delayed sleep onset and dysregulated melatonin secretion are prevalent and may be linked to mitochondria [149,150]. Functional imaging studies have revealed hypometabolism in the prefrontal cortex of ADHD patients, further supporting the hypothesis of altered metabolic processes [83,84].

Gastrointestinal disturbances are another feature commonly reported in autism and ADHD and may be linked to mitochondrial dysfunction [151]. The gastrointestinal tract requires substantial energy for motility, nutrient transport, and maintenance of epithelial integrity. Mitochondria in enterocytes play a central role in managing oxidative stress and regulating inflammatory responses, and disruptions in these processes may contribute to gastrointestinal symptoms [152]. Mitochondrial impairment within this system can lead to slowed motility, nausea, constipation, and altered nutrient absorption. Several studies describe histological abnormalities in the intestinal tissue of autistic children, including the reduced activity of enzymes involved in mitochondrial metabolism [153,154,155]. Gastrointestinal symptoms are also reported in ADHD, including constipation, incontinence, abdominal pain, dyspepsia, and irritable bowel syndrome [156,157]. While mitochondrial dysfunction is well known to contribute to the issue, the cause of gastrointestinal distress in both autism and ADHD is likely multifactorial and includes diet, sleep, stress, behavioral factors, gut–brain interactions, gut microbiota, and medication use [158].

Neurological features associated with mitochondrial dysfunction also appear in some individuals with autism and ADHD. Mitochondria play a critical role in regulating neuronal excitability and maintaining calcium homeostasis. When mitochondrial function is compromised, neurons become more susceptible to hyperexcitability and oxidative injury, which increases the likelihood of seizure activity [159,160]. Approximately 11–12% of autistic individuals and 6–14% of those with ADHD present with seizures [159,160,161]. Secondary mitochondrial dysfunction, including impairments in oxidative phosphorylation, increased oxidative stress, and disrupted redox balance, is implicated in seizure susceptibility and, conversely, is worsened by recurrent seizures, which can further impair mitochondrial function and worsen neuronal excitability [162].

Developmental regression is another clinical phenomenon that may intersect with mitochondrial dysfunction. Some autistic children experience a loss of previously acquired language, social, or motor skills, often during the second year of life [163,164]. A subset of these individuals demonstrate biochemical or genetic markers of mitochondrial impairment. Regression may reflect a period during which the energetic demands of the developing brain exceed the capacity of compromised mitochondria, leading to disruptions in synaptic stability and developmental plasticity. Elevated Complex IV hyperactivity reported in some cases of regressive autism further supports the idea that mitochondrial stress responses may play a role in developmental loss [45,164]. Although regression has multiple potential causes, mitochondrial involvement provides a plausible biological mechanism that warrants additional investigation. Note that developmental regression is largely specific to autism and is not a core feature of ADHD.

The clinical heterogeneity observed in autism and ADHD suggests that mitochondrial dysfunction may contribute to biological subtypes with distinct physiological profiles. Not all individuals with these conditions exhibit mitochondrial abnormalities, but those who do often present with recognizable constellations of symptoms [14,165]. These symptom clusters, including fatigue, gastrointestinal disturbances, motor challenges, regression and increased oxidative stress, may serve as indicators of underlying mitochondrial vulnerability [104,142,151,155]. Identifying these subgroups is essential for developing more personalized approaches to diagnosis and intervention, as individuals with mitochondrial involvement may respond differently to treatments targeting metabolic or oxidative pathways.

## 10. Neuroimaging Evidence of Altered Energy Metabolism

Neuroimaging provides an important perspective on mitochondrial dysfunction in autism and ADHD by revealing alterations in brain metabolism that rely on oxidative phosphorylation. Although these techniques do not measure mitochondrial activity directly, changes detected through magnetic resonance spectroscopy (MRS), positron emission tomography (PET), and functional magnetic resonance imaging (fMRI) often reflect underlying bioenergetic deficits. Across studies, these findings suggest that mitochondrial dysfunction contributes to atypical neural development in autism and may influence attentional and executive difficulties in ADHD [34,166].

Proton MRS offers key evidence by measuring metabolites linked to mitochondrial activity. Elevated lactate in the frontal lobes, basal ganglia, and cerebellum indicates a shift toward anaerobic metabolism, while reduced N-acetylaspartate in frontal and temporal regions points to impaired neuronal integrity [145,167]. Likewise, abnormalities in brain energy metabolism detected by ^31^P MRS, including reduced phosphocreatine to ATP ratios and altered high-energy phosphate levels, have been observed in neuroimaging studies of autism [63]. Together, these abnormalities suggest reduced mitochondrial efficiency in circuits involved in social cognition, communication, and sensory processing. PET studies complement these results by identifying altered glucose metabolism in autism, with frequent reports of hypometabolism in prefrontal, temporal, and cingulate areas that support social and executive function [168,169]. Some individuals show increased metabolic activity in sensory regions, possibly reflecting compensatory changes. Similar patterns appear in ADHD, where reduced glucose metabolism in prefrontal and striatal regions aligns with difficulties in attention, working memory, and cognitive control [138]. fMRI often detects unstable activation in these networks, which may reflect bioenergetic vulnerability affecting neurotransmitter systems. Structural imaging adds further support. Altered cortical growth, reduced white matter integrity, and atypical development of major tracts have been observed in autism [170,171] while delayed cortical maturation, reduced total brain volume, and alterations in frontostriatal and frontoparietal networks are reported in ADHD [34,172,173]. Because myelination and synaptic refinement are energy intensive, mitochondrial impairment during development may disrupt connectivity patterns common in neurodevelopmental conditions.

## 11. Biomarkers of Mitochondrial Function in Autism and ADHD

Biomarkers of mitochondrial function provide an important window into the physiological processes that underlie autism and ADHD. Because direct assessment of brain mitochondrial activity is rarely feasible, researchers rely on peripheral tissues, metabolic assays, and molecular indicators to identify signatures of mitochondrial dysfunction, [117,174]. These biomarkers offer insights into systemic metabolic health and often reflect broader physiological disturbances that may influence neurodevelopmental outcomes. Although no single biomarker definitively characterizes mitochondrial dysfunction in these conditions, similar patterns across studies reinforce the idea that mitochondrial impairment is present in a meaningful subset of individuals (Table 1).

### 11.1. Blood Biochemistry

One of the most frequently studied biomarkers in autism is lactate, a metabolite that increases when cells rely on anaerobic glycolysis due to inadequate oxidative phosphorylation [145]. Elevated lactate levels have been reported in blood, cerebrospinal fluid, and urine in a proportion of individuals with autism [145]. These elevations are often modest but consistent and may reflect chronic subclinical mitochondrial inefficiency. Some studies show that lactate levels rise further following physical exertion, which supports the presence of impaired mitochondrial responsiveness to metabolic demand [175]. Elevated pyruvate, particularly when observed alongside increased lactate, provides additional evidence for abnormalities in pyruvate metabolism and its entry into the tricarboxylic acid cycle [146]. The lactate-to-pyruvate ratio has been used to assess potential disruptions in electron transport chain function, with higher ratios suggesting impaired oxidative phosphorylation.

Blood-based biomarkers are less studied in ADHD. A systematic review of metabolomic markers in ADHD has been performed by Predescu and colleagues [69]. Five major metabolic pathways were found to be disrupted: oxidative and nitrosative stress, fatty acid metabolism, amino acid metabolism, neurotransmitter metabolism, and the kynurenine pathway. Like autism, lactate levels relative to controls are elevated, indicating a shift toward anaerobic glycolysis [69]. This finding is in line with the energy deficit hypothesis of ADHD, which proposes that reduced cerebral lactate availability from astrocytes contributes to symptomology. Elevated baseline levels of pyruvate and TCA cycle intermediates, including alpha-ketoglutarate and fumarate, have also been reported in individuals with ADHD, further implicating disrupted mitochondrial substrate metabolism [70].

### 11.2. Oxidative Stress

Markers of oxidative stress serve as another category of mitochondrial-related biomarkers. Increased levels of lipid peroxidation products, such as malondialdehyde and 4-hydroxynonenal, reflect oxidative damage to cell membranes [176,177]. Elevated concentrations of protein carbonyls and nitrotyrosine indicate protein oxidation and nitration, respectively. These biomarkers are frequently elevated in individuals with autism and are often accompanied by signs of reduced antioxidant capacity. Lower levels of reduced glutathione and altered glutathione redox ratios further support the presence of oxidative imbalance [49,50]. Antioxidant capacity also appears to be compromised in ADHD. Decreased total antioxidant status and lower glutathione peroxidase activity have been reported in children with ADHD [73]. Elevated oxidized glutathione disulfide, reflecting depletion of reduced glutathione, correlates positively with hyperactivity severity [75], while increased erythrocyte glutathione levels suggest that oxidative stress contributes to neuroinflammation [76]. Lipid peroxidation is another consistent finding. Elevated malondialdehyde has been reported in both adults [71] and children [72] with ADHD, indicating oxidative damage to cell membranes. Urinary acrolein-lysine levels are similarly elevated in affected children relative to controls. In sum, these disruptions in redox homeostasis provide indirect evidence of mitochondrial dysfunction given the central role of mitochondria in regulating cellular oxidative balance.

### 11.3. MtDNA Copy Number

MtDNA represent another category of biomarker that has gained attention in recent years. Variations in mtDNA copy number have been reported in autism with inconsistent findings across tissue types. In autism, a systematic review of six studies measuring blood mtDNA copy number (peripheral blood, leukocytes, and peripheral blood mononuclear cells) showed a small but non-significant increase compared to controls. In contrast, examination of buccal and brain tissue showed a significant increase in mtDNA copy number in autism compared to healthy controls [59]. Although mtDNA copy number is most commonly measured in peripheral blood, tissue-specific differences exist, with brain tissue showing distinct patterns that may not be reflected in blood samples, suggesting greater variability in blood measurements but more consistent elevation in brain and buccal tissues.

Elevations in mtDNAcopy number appear to be more consistent in ADHD. A systematic review and meta-analysis showed elevated mtDNA copy number in the peripheral blood of ADHD patients compared with healthy controls [59]. Increased copy number has been associated with hypomethylation of PPARGC1A, a transcriptional regulator of mitochondrial biogenesis and likely contributor to elevated mtDNA copy number [78]. Associations between mtDNA copy number and monoaminergic gene variants, including monoamine oxidase A (MAOA) and serotonin (5-HTT) polymorphisms, have also been identified, indicating a potential intersection between mitochondrial dysregulation and catecholamine neurotransmitter metabolism in ADHD [79]. A one-year longitudinal follow-up study found that while absolute mtDNA copy number remained stable over time regardless of treatment, reductions in mtDNA copy number correlated with decreased inattention severity specifically in the treated group, suggesting that symptomatic improvement with pharmacotherapy may track with normalization of mitochondrial activity [80].

### 11.4. MtDNA Haplogroups, Heteroplasmy and Deletions

mtDNA variation in neurodevelopmental disorders is best understood across three mechanistically related levels: inherited haplogroups, somatic heteroplasmy, and deletions. Haplogroup-determined differences in mitochondrial efficiency likely establish a bioenergetic baseline that influences susceptibility to somatic mtDNA damage, while heteroplasmy reflects progressive declines in mtDNA integrity. Certain mtDNA haplogroups may increase autism risk [178]. Experimental evidence demonstrates that haplogroups exhibit functional differences in oxidative phosphorylation efficiency [179,180], which may underlie differential disease susceptibility. mtDNA haplogroups are also associated with both increased and decreased ADHD risk [81,82]. Collectively, these associations suggest mitochondrial bioenergetic contributions to neurodevelopmental conditions.

Heteroplasmy, the coexistence of mutant and wild-type mtDNA molecules within a cell, represents another important source of mtDNA variation in autism. Autistic individuals harbor 53% more heteroplasmic mutations at non-polymorphic mtDNA sites compared to their siblings [181]. These findings suggest that accumulation of low- to medium--level pathogenic mtDNA heteroplasmies during development may contribute to autism risk. Comparable heteroplasmy data for ADHD could not be found. Lastly, large-scale deletions, arising through oxidative damage and replication errors, represent the most severe form of mtDNA structural damage and occur more frequently in autism than in healthy controls [101]. They are important because they can reduce oxidative capacity and increase ROS production, creating a self-perpetuating cycle that is detrimental to synaptic and neurodevelopmental abnormalities, especially during critical windows. These mutations often co-exist with nuclear variants, suggesting intergenomic coordination. We are not aware of reports documenting mtDNA deletions in ADHD.

### 11.5. Other Markers

Respiratory chain enzyme activities measured in peripheral tissues provide a more direct assessment of mitochondrial function. Buccal swab assays, lymphocyte enzyme analyses, and muscle biopsies have revealed reduced activity in one or more electron transport chain complexes in a subset of individuals with autism [113,117,118]. Complex I deficiency is the most consistently reported abnormality and often co-occurs with signs of oxidative stress. Elevated Complex IV activity has also been reported in some individuals, particularly those with developmental regression [164]. Similar studies on ADHD could not be found.

## 12. Conclusions

Evidence from genetics, biochemistry, and brain imaging consistently points to mitochondrial dysfunction as a biological feature of autism and, to a lesser degree, ADHD. The most replicated findings (e.g., reduced Complex I activity, elevated lactate, lower glutathione levels, and altered mtDNA copy number) are not present in all affected individuals, suggesting that mitochondrial involvement may define a subgroup rather than the condition as a whole.

Whether mitochondrial dysfunction acts as a primary driver, a downstream consequence of oxidative and inflammatory stress, or a modifier of clinical course remains unresolved. Cross-sectional study designs hinder causal inference, a significant limitation across the neurodevelopmental literature. There is a clear need for standardized diagnostic criteria for secondary mitochondrial dysfunction in neurodevelopmental conditions to enable reliable patient stratification. Without consistent definitions, comparing findings across studies and identifying which individuals are most likely to benefit from mitochondria-targeted approaches remains difficult. Developing these criteria is a necessary step toward translating these findings into clinical practice.

## Figures and Tables

**Table 1:** Biomarkers of mitochondrial dysfunction in autism and ADHD. Review of various physiological and molecular indicators associated with impaired mitochondrial function observed in autistic individuals. Biomarkers are categorized by the pathway they reflect, detailing the observation, sample source, and supporting citations.

Biomarker Category	Biomarker Details	Observations	Type/Tissue	Studies
**Autism**				
Metabolic/Energy substrates	Lactate ↑, lactate/pyruvate ratio ↑	Increased reliance on anaerobic glycolysis due to impaired oxidative phosphorylation	Blood, CSF	Coleman & Blass (1985) [41]; * Frye (2024) [8], * Rossignol & Frye (2012) [42], *
ETC function (Protein/Activity)	Complex I activity ↓ Complex V activity ↓ Regional ETC deficits in frontal cortex, cerebellum, and temporal cortex Occasional ↑ in Complex IV activity	Most commonly affected complex; reduced function in brain regions Observed in a subset, potentially a maladaptive response.	Postmortem Brain (Temporal Cortex, Frontal Cortex, Cerebellum), Muscle, Buccal Cells	Gu et al. (2013) [4]; Chauhan et al. (2011) [43]; Rossignol & Frye (2012) [42] Palmieri et al. (2010) [44]; Frye & Naviaux (2011) [45]; Ginsberg et al. (2012) [46]
TCA Cycle/Enzyme	Pyruvate Dehydrogenase (PDH) activity ↓ ↓ aconitase activity	Reduced link between glycolysis and the TCA cycle in the frontal cortex.	Postmortem Brain (Frontal Cortex).	Gu et al. (2013) [4]; Chauhan et al. (2011) [43], * Frye et al. (2024) [8].
Oxidative Stress	Lipid Hydroperoxides (LOOH) ↑ 3-Nitrotyrosine (3-NT) ↑ Malondialdehyde (MDA) ↑ Nitric oxide ↑	Marker of lipid peroxidation/oxidative damage. Marker of protein nitration/oxidative damage.	Brain (Temporal Cortex, Cerebellum)	Chauhan et al. (2011) [43]; Tang et al. (2013) [47]; Evans et al. (2008) [48] Sajdel-Sulkowska et al. (2011) [49]; Rose et al. (2012) [50], * Chen et al. (2021) [51]
Redox state/antioxidants	Glutathione (GSH) ↓ GSH:GSSG Ratio ↓ Vitamin B9 (folate), Vitamin B12, Vitamin D, Vitamin E ↓ Methionine,↓ Cysteine ↓ S-adenosylmethionine/S-adenosylhomocysteine ratio ↓	Shift toward an oxidative cellular state.	Blood, Brain (Cerebellum, Temporal Cortex)	James et al. (2004, 2006) [52,53]; Rossignol & Frye (2014) [54], * Chen et al. (2021) [51]
mtDNA (Genomic)	mtDNA Copy Number ↑ mtDNA Deletions (ND4, Cyt B) Heteroplasmy	Potentially compensatory response to low ETC activity. Presence of deleted mtDNA in ETC subunit genes. Associated with greater autism score severity in some cohorts. Suggests compensatory mitochondrial biogenesis and mitochondrial genomic instability.	Brain (Frontal cortex), Blood	Giulivi et al. (2010) [55] Pons et al. (2004) [56], Caporali et al. (2022) [57]; Wang et al. (2022) [58], * Al-Kafaji et al. (2023) [59]
Mitochondrial dynamics/Mitophagy	Mitochondrial fission proteins ↑ fusion proteins ↓ Pink1/Parkin mitophagy markers ↑ Pgc-1α and Tfam ↓	Altered mitochondrial quality control, impaired biogenesis, and disrupted mitochondrial turnover.	Human tissue, mouse models	Tang et al. (2013) [47]; Khacho et al. (2022) [31]; Co et al. (2020) [60]; Wang et al. (2022) [22]
Metabolic byproducts Metabolic substrates	Alanine ↑, Ammonia ↑ Carnitine (Free/Total) ↓ Creatine Kinase ↑; AST ↑	Imbalance in amino acid metabolism/detoxification, reflecting inability to shuttle fatty acids into mitochondria. Imbalance in amino acid metabolism/detoxification. Elevated prevalence of alanine and creatine kinase in ASD, some also had elevated AST.	Plasma, Serum	Frye (2020) [61], Filipek et al. (2004) [62]; * Rossignol & Frye [42], * Frye et al. (2024) [8]
Stress markers (systemic)	FGF21/GDF15 ↑	Hormones secreted in response to mitochondrial stress.	Serum, CSF	Frye et al. (2020) [61], * Frye et al. (2024) [8]
Neuroimaging (MRS)	N-Acetylaspartate (NAA) ↓ PCr + Cr ↓/GPC + PC ↓ GABA ↓; Choline (Cho) ↓ Glutamate (Glu) ↑ in prefrontal cortex	Markers of neuronal integrity and mitochondrial function in neurons. Compromised high-energy phosphate reserves. GABA reductions most pronounced in limbic regions	Brain (Thalamus, Gray/White Matter, Frontal/Temporal Cortices)	Minshew et al. (1993) [63], * Du et al. (2023) [64], * Thomson et al. (2024) [65]
Inflammation (brain/peripheral)	Microglial Activation/IL-6 ↑ IL-1β, TNF-α, IFN-γ, IL-12, MIF, eotaxin-1 (blood) ↑ Anti-inflammatory cytokines (IL-10, TGF-β) in peripheral blood ↓	Indicates chronic neuroinflammation that impacts neuronal function pro-inflammatory profile in ASD with concurrent anti-inflammatory deficit Peripheral cytokine findings complement postmortem brain microglial activation data, supporting a systemic neuroinflammatory state.	Postmortem Brain (Cerebellum, Frontal/Temporal Cortex), Blood (serum/plasma)	Vargas et al. (2005) [66]; Morgan et al. (2010) [67], * Zhao et al. (2021) [68]
**Attention-Deficit/Hyperactivity Disorder**				
Metabolic/Energy substrates	Lactate ↑ Pyruvate ↑ TCA intermediates ↑ (alpha-ketoglutarate, fumarate)	Shift toward anaerobic glycolysis Elevated baseline pyruvate and TCA intermediates indicate disrupted mitochondrial substrate metabolism.	Blood, Plasma	* Predescu et al. (2024) [69], Ellis et al. (2025) [70]
ETC function (Protein/Activity)	ETC Complex I activity ↓ Complex V activity ↓ Mitochondrial membrane potential ↓ ATPase 6/8 transcript levels ↓	Cybrid cell lines from ADHD patient platelets. Show reduced Complex I and V activities and loss of mitochondrial membrane potential.	Cybrid cell lines	Verma et al. (2016) [9]
Oxidative Stress	Malondialdehyde (MDA) ↑ Urinary acrolein-lysine ↑ Total antioxidant status ↓ Glutathione peroxidase activity ↓	Elevated MDA in both children and adults with ADHD; oxidative membrane damage Reduced antioxidant defenses.	Blood Urine	Bulut et al. (2013) [71], Ceylan et al. (2010) [72], Sezen et al. (2016) [73], * Corona JC (2020) [74]
Redox state/Antioxidants	Oxidized glutathione (GSSG) ↑ Erythrocyte GSH ↑ GSH:GSSG ratio altered	Elevated GSSG reflects depletion of reduced GSH; correlates positively with hyperactivity severity Meta-analysis: normal antioxidant production but insufficient response to oxidative challenge, resulting in net oxidative damage	Blood	Dvořáková et al. (2007) [75], Verlaet et al. (2019) [76], * Joseph et al. (2015) [77]
mtDNA Copy Number	mtDNA copy number ↑ (peripheral blood) PPARGC1A hypomethylation MAOA/5-HTT variant associations Longitudinal: copy number reduction tracks treatment response	Elevated mtDNA copy number in peripheral blood vs. healthy controls (meta-analysis) Increased copy number associated with hypomethylation of PPARGC1A (mitochondrial biogenesis regulator) Associations with gene variants for catecholamine metabolism Reduction in copy number correlates with decreased inattention severity in treated group at 1-year follow-up	Peripheral blood, Leukocytes	* Al-Kafaji et al. (2023) [59], Kim JI et al. (2019) [78], Citrigno L et al. (2025) [79], * Gawali et al. (2023) [80]
mtDNA Haplogroups	Haplogroup risk associations (increased and decreased ADHD risk)	Certain mtDNA haplogroups are associated with both increased and decreased ADHD risk Haplogroup-determined differences in OXPHOS efficiency may influence neurodevelopmental susceptibility	Blood	Chang et al. (2020) [81], Hwang IW et al. (2019) [82]
Neuroimaging (Functional)	Hypometabolism in prefrontal cortex Reduced glucose metabolism in prefrontal and striatal regions	Functional imaging reveals prefrontal and striatal hypometabolism in ADHD; supports bioenergetic vulnerability in attentional and executive networks	Brain (fMRI, PET)	Salavert et al. (2018) [83], Chen et al. (2025) [84]
Neuroinflammation (peripheral/systemic)	Elevated serum cytokines Above-chance co-occurrence with autoimmune/inflammatory disorders Polymorphisms in inflammation-related genes	Evidence largely peripheral; no postmortem human brain studies of glial activation exist for ADHD. Findings primarily from comorbidity data and serum cytokine reports	Blood (serum), Genetic	Leffa et al. (2018) [85], Dunn et al. (2019) [86], * Corona JC (2020) [74]

* Indicates systematic review and/or meta-analysis; ↑ and ↓ show direction of change.

## Data Availability

No new data were created or analyzed in this study. Data sharing is not applicable to this article.

## Supplementary Materials

The following supporting information can be downloaded at https://www.mdpi.com/article/10.3390/antiox15060764/s1, File S1: Search strategy.
