# Supplementary material for: Mitochondrial Dysfunction in Autism and Attention-Deficit/Hyperactivity Disorder: Evidence from Genetic, Biochemical, and Neuroimaging Approaches
Source: Antioxidants (Basel). 2026 Jun 18;15(6):764. doi: 10.3390/antiox15060764 (PMC13295364; doi:10.3390/antiox15060764)
Supplement: Supplementary file 1 [file antioxidants-15-00764-s001.zip › antioxidants-4299708-supplementary/Search_Strategy_MDPI (2).docx]

**Detailed Search Strategy**

*Study Design & Literature Search Strategy.* Conducted in accordance with the Scale for the Assessment of Narrative Review Articles (SANRA) ^1^ . A comprehensive literature search was performed using PubMed (MEDLINE) and Google Scholar to identify relevant studies published between January 1968 and March 2025. Earlier foundational studies were included to provide historical context.

*Search Structure.* The PubMed search strategy was structured around four themes: (1) ASD and ADHD, (2) mitochondrial biology, (3) metabolism, and (4) mechanistic pathways such as oxidative stress, bioenergetics, and neuroinflammation. Boolean operators (AND/OR) were used where appropriate. (Autism Spectrum Disorder OR ASD OR ADHD) AND (mitochondria OR mitochondrial dysfunction OR bioenergetics) AND (metabolism OR metabolic) AND (oxidative stress OR mtDNA OR ROS OR neuroinflammation OR metabolism). In addition, Google Scholar was used to capture relevant studies not catalogued by PubMed keywords such as 'mitochondrial dysfunction, autism, ADHD', 'mtDNA autism heteroplasmy', and 'brain lactate autism MRS'.

*Inclusion and Exclusion Criteria.* Abstracts were screened and studies that examined mitochondrial function in ASD/ADHD, including genetic, biochemical, neuroimaging, or clinical evidence were catalogued. Human studies in were prioritized over animal studies. Likewise, studies in children of developmental age were of particular interest. Studies not related to neurodevelopmental conditions, non-mitochondrial mechanisms, non-English publications, those lacking relevance or that repeated findings found elsewhere were excluded.

*Data Synthesis.* Findings were synthesized qualitatively and grouped into themes including genetics, biochemistry, oxidative stress, neuroinflammation, neuroimaging, and biomarkers.

*Search Data*

| **IDENTIFICATION** |  |
| --- | --- |
| Records identified through PubMed search: | n ≈ 345 |
| Records identified through Google Scholar: | n ≈ 42 |
| Additional records from reference lists: | n ≈ 34 |
| Removal of Duplicates | n ≈ -18 |
|  | **n ≈ 403** |
| **SCREENING** |  |
| Records screened (title/abstract): | n ≈ 403 |
| Records excluded: - Not mitochondrial-focused; not ASD/ADHD-related; irrelevant outcomes; non-English or French, case studies. | n ≈ 133 |
|  | **n ≈ 270** |
| **ASSESSED** |  |
| Full-text articles assessed: | n ≈ 270 |
| Full-text articles excluded: Non-human (unless mechanistically critical), redundant, low quality evidence, speculative. | n ≈ 90 |
| Included: Studies included in narrative synthesis: n ≈ 230 | **n ≈ 180** |
