# Supplementary material for: Mitochondrial Dysfunction in Autism and Attention-Deficit/Hyperactivity Disorder: Evidence from Genetic, Biochemical, and Neuroimaging Approaches
Source: Antioxidants (Basel). 2026 Jun 18;15(6):764. doi: 10.3390/antiox15060764 (PMC13295364; doi:10.3390/antiox15060764)
Supplement: Supplementary file 1 [file antioxidants-15-00764-s001.zip › antioxidants-4299708-supplementary/Supplementary File List (1).docx]

*Antioxidants Special Issue: Oxidative Stress, Mitochondrial Dysfunction, and Inflammation in Autism.*

**Secondary Mitochondrial Dysfunction in Autism and Attention-deficit Hyperactivity Disorder: Evidence from Genetic, Biochemical, and Neuroimaging Approaches**

Ram et al.

**Supplementary Files Included in Submission**

- SARNA Checklist
- Search Strategy
- Biorender.com Publishing Licences
